# Supplementary material for: Supersampled Scanning Transmission X‐Ray Microscopy for High‐Resolution Vibration‐Independent Time‐Resolved Imaging
Source: Small. 2026 Jul 25;22(50):e74569. doi: 10.1002/smll.74569 (PMC13548941; doi:10.1002/smll.74569)
Supplement: Supplementary file 1 [file SMLL-22-e74569-s001.pdf]

Supporting Information

Supersampled scanning transmission X-ray microscopy for high-resolution vibration independent time-resolved imaging

Simone Finizio,[1,*] Benjamin Watts,[1] Benedikt Rösner,[1] Tim A. Butcher,[2] Sebastian Wintz,[3] Markus Weigand,[3] and Jörg Raabe[1]

[1]*Paul Scherrer Institut, 5232 Villigen PSI, Switzerland*

[2]*Max Born Institute for Nonlinear Optics and Short Pulse Spectroscopy, 12489 Berlin, Germany*

[3]*Helmholtz Zentrum Berlin für Materialien und Energie, 12489 Berlin, Germany*

(Dated: May 4, 2026)

I. SETUP OF THE PANDABOX FPGA

The FPGA of the PandABox data acquisition tool was programmed using the native software of the device, which is structured in separate functional blocks that can be connected together. For the work described here, the following blocks and connections were utilized (refer to the PandABlocks manual [1] for details on the specific block names).

- CLOCK1: A 4 kHz clock was configured using the built-in PandABlocks CLOCK block. The clock is always enabled.
- INENC1/2: Two blocks handling the interferometer input, based on the built-in PandABlocks INENC block.
- PCAP: Block handling the position capture. The block is always enabled and triggered by the 4 kHz CLOCK1 block.
- TTLIN1: TTL input block, to which the output of the photon counting unit is connected to.
- TTLIN2: TTL input block, to which a digital signal set to active when an image is being acquired is connected to.
- COUNTER1: A PandABlocks COUNTER block, enabled by the TTLIN2 input, incremented by the TTLIN1 signal.

The captured position data, together with the COUNTER1 value, is streamed from the PandABox with a rate defined by CLOCK1.

II. DISTRIBUTION OF DWELL TIMES

The binning process described in the main manuscript produces images with a non-constant pixel dwell time, due to the fact that the positioning requirement precision is significantly relaxed if compared to an equivalent constant velocity or point-by-point scan. This implies that the recorded photon counts per pixel need to be normalized by the actual pixel dwell time, and that the quantity that will be displayed in the final image is the

* simone.finizio@psi.ch

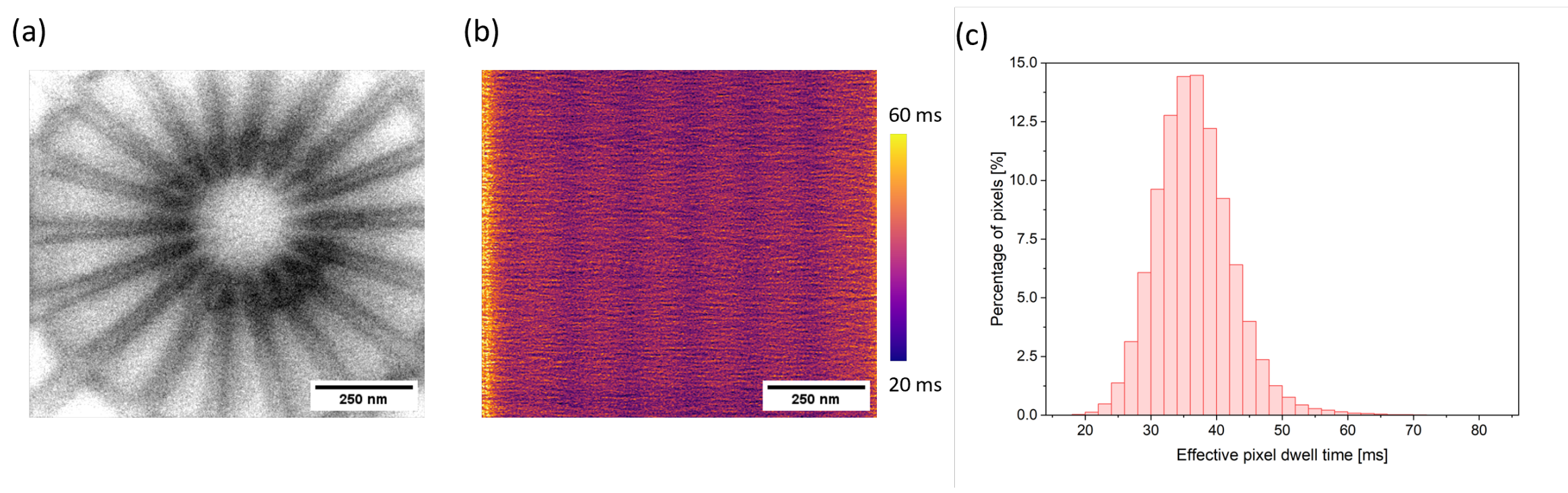

FIG. S1. (a) Supersampled microscopy image of the center region of a 10 nm Ir Siemens star. (b) Map of the total dwell time per pixel. (c) Histogram of the data shown in (b), showing a distribution of dwell times with a mean value of about 38 ms and a standard deviation of about 15 ms.

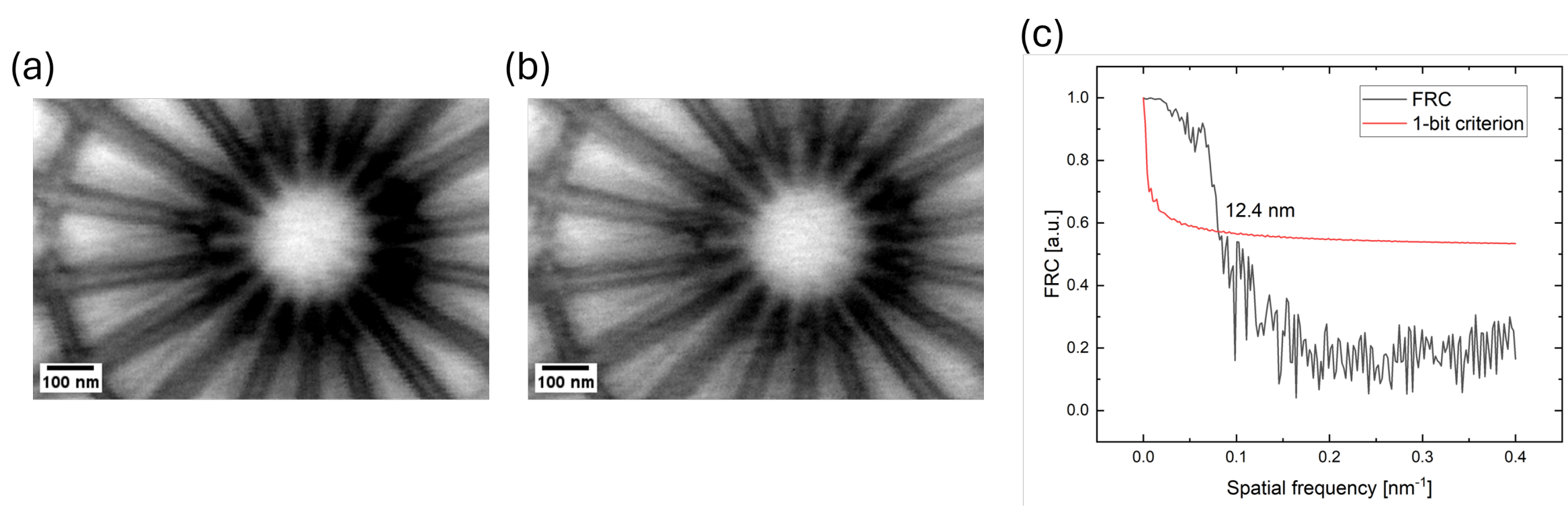

FIG. S2. High resolution images acquired with the supersampled microscopy method. (a-b) Two consecutive images of a 10 nm Ir Siemens star reconstructed with a pixel size of 2.5 nm. (c) Fourier ring correlation calculated from (a-b), with a 1-bit criterion resolution of 12.4 nm.

photon count rate. The distribution of pixel dwell times in the image depends on several parameters, which affect how the scanning is performed (e.g. scan trajectory, velocity of the stages, etc.). For the scan shown in Fig. 5 of the main manuscript, the distribution of dwell times is displayed in Fig. S1, with a mean pixel dwell time of 38 ms and a standard deviation of 15 ms. Calculations of the Poisson noise will have to take this into consideration.

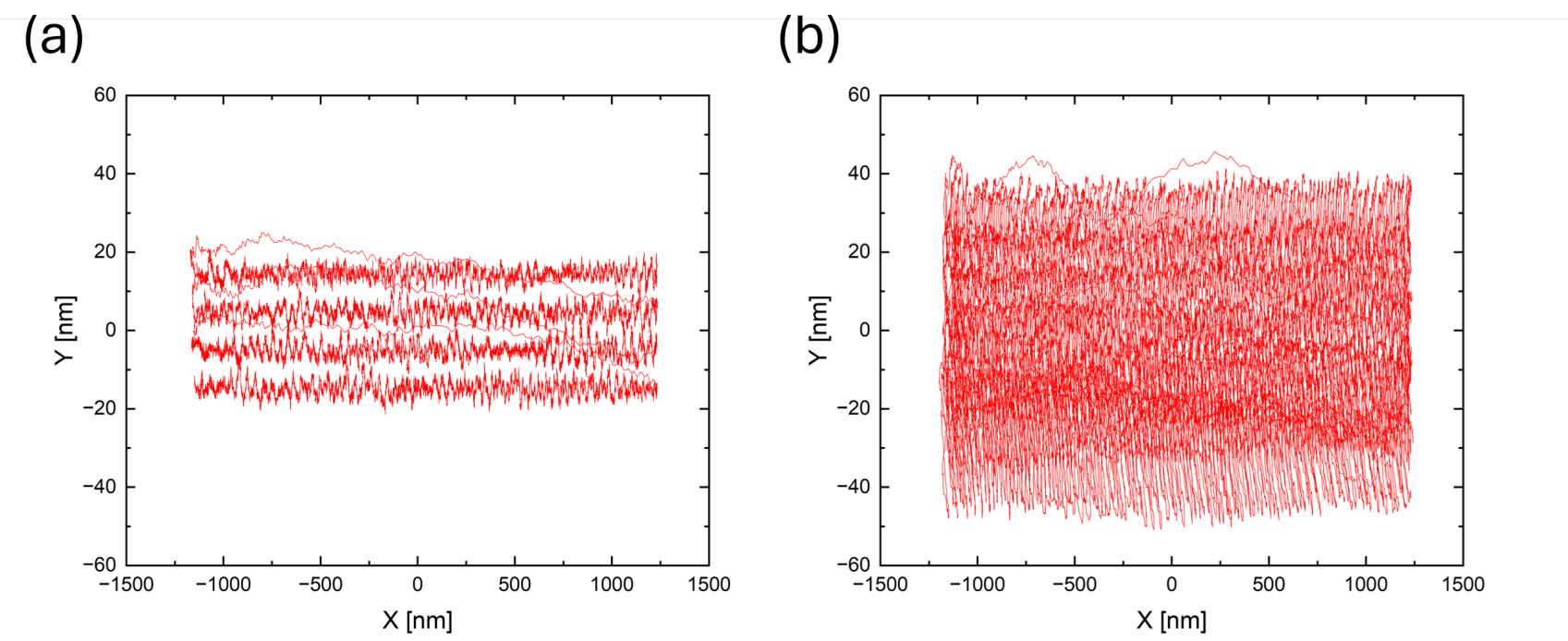

FIG. S3. Effect of mechanical vibrations on the sampling. (a) Trace of the position of the sample in a 4-line scan under low vibration conditions. Several regions between the lines are not sampled. (b) Same 4-line scan under high vibration conditions. The mechanical vibrations lead to a more uniform sampling of the region of interest.

III. ADDITIONAL HIGH-RESOLUTION IMAGES

In this section, additional high-resolution STXM images acquired with the supersampled imaging protocol are shown, together with the calculation of the spatial resolution through Fourier ring correlation (FRC) [2] between two equal scans acquired sequentially. The images of a 10 nm Ir Siemens star shown in Fig. S2 have been acquired with a 18 nm outermost zone width zoneplate in diffraction limited conditions, and the FRC calculation shows that the theoretical limit of the optics is reached.

IV. VIBRATION-ASSISTED SAMPLING

In this section, we show the effect of mechanical vibrations on the sampling of a region of interest, depicted in Fig. S3 with a subset of the data used to reconstruct Fig. 5 in the main manuscript. In Fig. S3(a), acquired under low vibration conditions, one can observe that the region of interest is not uniformly sampled by the beam. Under high vibration conditions (Fig. S3(b)), the region of interest is sampled more uniformly. Mechanical vibrations can therefore be harnessed to allow for a uniform sampling of a region of interest without the

requirement to perform a dense scanning.

---

[1] Pandablocks documentation, `https://pandablocks.github.io/PandABlocks-FPGA/master/blocks.html`, accessed: 2025-12-03.

[2] N. Banterle, K. H. Bui, E. A. Lemke, and M. Beck, Fourier ring correlation as a resolution criterion for super-resolution microscopy, Journal of Structural Biology **183**, 363 (2013).
